# A Remote Calibration Device Using Edge Intelligence

**DOI:** 10.3390/s22010322

**Published:** 2022-01-01

**Authors:** Quan Wang, Hongbin Li, Hao Wang, Jun Zhang, Jiliang Fu

**Affiliations:** 1 School of Electrical and Electronic Engineering, Huazhong University of Science and Technology, Wuhan 430074, China; lihongbin@hust.edu.cn; 2 China Electric Power Research Institute Co., Ltd., Wuhan 430074, China; Zhangjun3@epri.sgcc.com.cn (J.Z.); Fujiliang@epri.sgcc.com.cn (J.F.); 3 State Key Laboratory of Advanced Electromagnetic Engineering and Technology, Wuhan 430074, China; 4 National Institute of Metrology, Beijing 100029, China; wh1967@nim.ac.cn

**Keywords:** remote calibration device, edge intelligence, text recognition, CRNN, power system

## Abstract

Power system facility calibration is a compulsory task that requires in-site operations. In this work, we propose a remote calibration device that incorporates edge intelligence so that the required calibration can be accomplished with little human intervention. Our device entails a wireless serial port module, a Bluetooth module, a video acquisition module, a text recognition module, and a message transmission module. First, the wireless serial port is used to communicate with edge node, the Bluetooth is used to search for nearby Bluetooth devices to obtain their state information and the video is used to monitor the calibration process in the calibration lab. Second, to improve the intelligence, we propose a smart meter reading method in our device that is based on artificial intelligence to obtain information about calibration meters. We use a mini camera to capture images of calibration meters, then we adopt the Efficient and Accurate Scene Text Detector (EAST) to complete text detection, finally we built the Convolutional Recurrent Neural Network (CRNN) to complete the recognition of the meter data. Finally, the message transmission module is used to transmit the recognized data to the database through Extensible Messaging and Presence Protocol (XMPP). Our device solves the problem that some calibration meters cannot return information, thereby improving the remote calibration intelligence.

## 1. Introduction

Traditional calibration requires an expert to operate personally in a scene which is rather inefficient. At the same time, the standard calibration equipment needs to be transported to the site. Obviously, this consumes a lot of time and transportation costs. Nowadays, most digital instruments and virtual instruments provide communication interfaces that makes it possible to form a remote calibration system based on actual hardware and computer units with network interconnection capabilities. In addition, with the rapid development of Internet and bus technology, making full use of the worldwide Internet for remote calibration has gradually received the attention of researchers [1,2,3]. Mellit A et al. [4] designed a novel prototype for remote monitoring of a greenhouse that can offer real-time remote measuring and sensing services to farmers. Gunawan TS et al. [5] designed a one power factor meter using Internet of Things and further discussed the analysis of the smart meter IoT framework that can provide power factor improvement, remote monitoring, and data logging.

In view of the above problem and cases, we propose a remote calibration device that incorporates edge intelligence. We only need to bring the self-developed portable heterogeneous network-based edge devices and standard parts to the site. The heterogeneous edge devices mainly include a wireless serial port module, a Bluetooth communication module, a video acquisition module, a text recognition module, and a message transmission module. We introduce a wireless serial port and Bluetooth communication to realize networked remote calibration. In particular, for the data of some specific meters in the remote calibration are not returned through the network, we introduce artificial intelligence technology to complete the recognition of these meters, and then transmit recognized data to the database of the central laboratory in real time through XMPP. We use a mini camera to capture images of the meters. Then, we adopt the EAST-based text detection and the CRNN-based text recognition to complete the recognition of meters. The experiments show that it has high recognition accuracy for some specific meters. At the same time, to ensure more efficient remote calibration, we use cameras to monitor the calibration laboratory. Then, we pull video streams through the video capture module, and transmit the video and dashboard pictures to the Android client of the central laboratory for inspecting whether the data calibration is wrong.

The contributions of our work are three-fold:

- We propose a remote calibration device that incorporates edge intelligence to improve the efficiency of calibration work and reduce costs;
- We combine a variety of heterogeneous edge devices to make remote calibration better networked;
- We propose a smart meter reading method in our device that is based on artificial intelligence to obtain information about calibration equipment and improves the intelligence.

This paper has the following structure and organization. Firstly, the previous cases and work of remote calibration are discussed in Section 2. Section 3 describes the overall architecture and functions of remote calibration that we designed. The main functional modules include wireless serial port, Bluetooth, XMPP, text recognition, and so on, are shown in Section 4. Section 5 presents the results of each functional module. In Section 6, we discuss the reason of model choosing, analyze the challenges and describe prospects for future work. Finally, in Section 7, we summarize our research results and the clarify significance of the work.

## 2. Related Work

Remote calibration can effectively solve many problems of traditional calibration, such as: high cost of sending inspection teams, long calibration period, inflexible sending time, etc. At present, the application of remote calibration in instruments and meters has received extensive attention. This section mainly focuses on works that are mostly relevant to the proposed materials and methods.

First, there are many technical research cases for remote calibration of different instruments. Wen Tian et al. [6] proposed a remote automatic detection method for AC energy meters based on cloud technology, wireless communication technology, and automatic control technology, which greatly saved the cost of manual calibration. Kai Han et al. [7] proposed a NIMDO-based time-frequency calibration method. This paper used NIMDO as the reference time-frequency source and calibrating NTP and rubidium atomic frequency by remote calibration, which better solved the problem of time traceability in the transportation field. Yuan-Long Jeang et al. [8] proposed a calibration method for servomotors to prevent the servomotors from being out of control, in this paper, a calibration control feedbacks system with remote monitoring features and parametric designs can be built rapidly through FPGA.

At present, several international institutions have conducted research related to remote calibration, for example, the US National Institute of Standards and Technology (NIST), the Federal Institute of Physics and Technology (FIPT), the National Measurement Institute of Japan (NMIJ), and the National Physical Laboratory (NPL) in the UK, etc. Their remote calibration projects are shown in Table 1. We can find that most of these studies explore non-physical signals (easily transmitted through the network) such as time, frequency, etc. Therefore, our research uses the Internet of Things technology to calibrate some physical signals and transmit them through the network, such as image information.

Second, scene text detection and recognition have been active research topics in computer vision for a long period of time. In particular, we have introduced scene text detection and recognition technology in the remote calibration process to enhance the intelligence. Comprehensive reviews and detailed analyses about the application of the scene text detection and recognition can be found in survey papers [9,10,11].

The traditional method of text detection and recognition is as follows: first, use binarization, skew correction, character cutting, etc., to preprocess the image. Second, recognize the cut characters by extracting artificially designed features, such as Histogram of Oriented Gradient (HOG) features and Convolutional Neural Network (CNN) feature maps. However, these methods fall behind those based on deep learning, in terms of both accuracy and adaptability, especially when dealing with challenging or complicated scenarios, such as low resolution and geometric distortion. For example, Baoguang Shi et al. [12] proposed a network structure: Convolutional Recurrent Neural Network (CRNN), which combined a Deep Convolutional Neural Network (DCNN) with a Recurrent Neural Network (RNN). It transformed image recognition into sequence recognition and achieved good recognition results.

In this paper, our main work is to apply different heterogeneous edge devices to help the central laboratory communicate with the calibration laboratory through the related network communication protocol. Firstly, we use a large camera to monitor the calibration process in the calibration laboratory. Then, the video screen under monitoring is transmitted to the server through the Real Time Messaging Protocol (RTMP) and displayed on the android client we designed in the central laboratory. RTMP is an application layer protocol, which based on TCP. It is an open protocol developed for audio, video and data transmission between Flash players and servers [13]. Secondly, we use a mini camera to face the meter to capture images, and then recognize it through our pre-trained text recognition model. Finally, we use specific text filtering methods by observing the image information of the meters to obtain useful data and transfer them to the database in central laboratory through XMPP. We adopt XMPP because it is based on the Jabber protocol, and Jabber is an open protocol commonly used in instant messaging. It inherits the flexible extensibility of the Extensible Markup Language (XML) environment [14,15]. The recognition of meters is the main work of this paper, and it is also the work that other researchers have not done before in the previous remote calibration. Thirdly, we use Bluetooth to connect our camera with other edge devices and transfer the data to the server, android client, and our computers. Bluetooth is a wireless data exchange technology, which supports short-distance communication of devices (generally within 10 m). It can be implemented between many mobile devices including mobile phones, wireless headsets, notebook computers, and related peripherals [16]. Finally, in the actual test, we will also encounter many difficulties and shortcomings. For example, the recognition effect of meters with unclear screens is poor. In addition, for meters with complex data information, it becomes very difficult to parse and extract useful data.

## 3. Overall Functional Architecture

**Figure 1:**
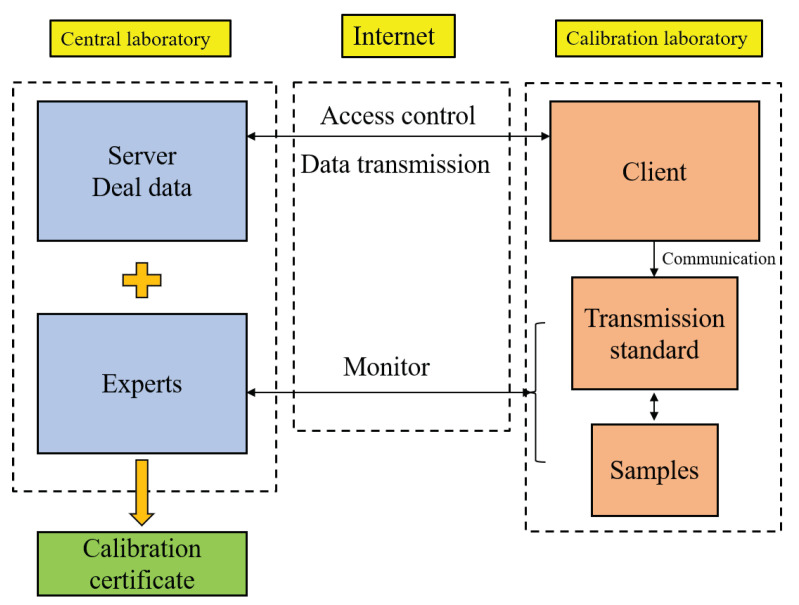
Remote calibration service system framework.

The remote calibration service mode should be performed by the central laboratory sending physical or non-physical standard signals to the calibrated laboratory and monitoring and operating the calibration process remotely, as shown in Figure 1.

The purpose of this paper is to build a heterogeneous network-based edge device for the remote calibration system to improve the intelligence of remote calibration work.

**Figure 2:**
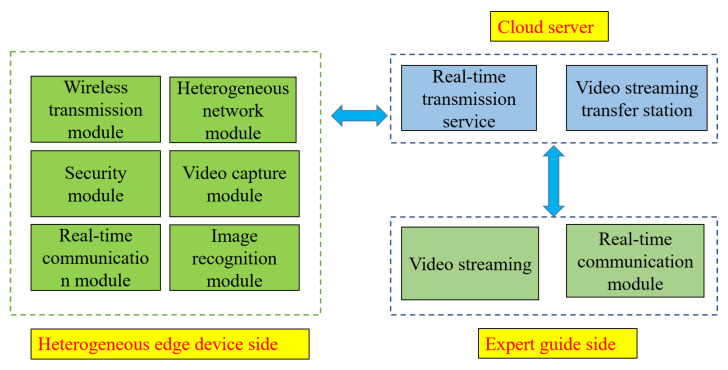
Main modules of the edge devices.

In practice, it is only necessary to bring our self-developed portable heterogeneous edge devices to the site to realize remote operation. The main functional modules of the edge device based on a heterogeneous network are shown in Figure 2.

These modules perform the following functions:

- Real-time video content transmission: video capture module needs to collect real-time video information and send it to the expert guide, and supports many-to-one. One expert guide can support multiple calibration processes based on it;
- Real-time sensor data transmission: the sensor information collected by heterogeneous network equipment should be transmitted to the expert guidance terminal in real time to facilitate the analysis of the calibration process. In this paper, it mainly refers to the meter information collected by the camera. In addition, there is some temperature and humidity information in the calibration laboratory;
- Character recognition of special electric meters: for the test data on some special electric meters, the camera is used to read the meter, the text recognition technology is used to identify the text and data in the meter, and the data need to be transferred to the database through XMPP. It communicates with the computer interactively and sends it to the database to complete the real-time collection and recording of test data.

**Figure 3:**
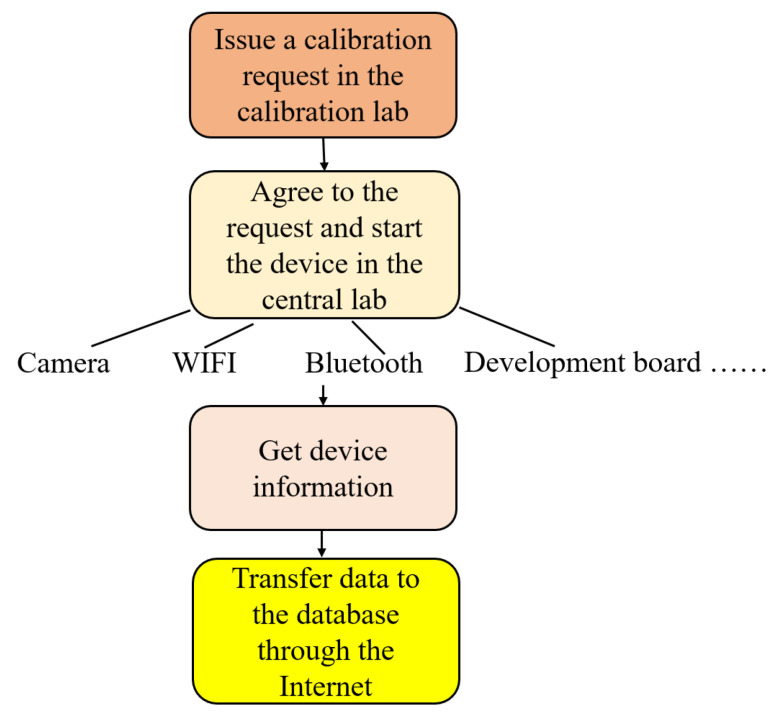
Step of remote calibration.

The realization of the overall function is carried out according to the following steps, as shown in the following schematic diagram in Figure 3.

In order to complete the calibration process in various scenarios, various heterogeneous network nodes, such as NBIoT [17], LoRa [18], Bluetooth, Zigbee [19], etc., may be required. In scenarios that require real-time data transmission, Bluetooth is more suitable for sensor data transmission than LoRa in the lab because of its lowest transmission power consumption and low latency. In addition, in scenarios where real-time video transmission is required, we can use Wi-Fi transmission because of its higher bandwidth. However, these heterogeneous nodes often use different communication front-ends and communication protocol stacks. Obviously, it is difficult. For this reason, a coprocessor is introduced to complete the protocol conversion. The coprocessor uses a unified interface to complete the information exchange with the smart terminal. In this way, after the software development of the smart terminal is completed, no additional adaptation work is needed. Adaptation and protocol conversion can be done on a low-cost MCU to reduce development workload.

**Figure 4:**
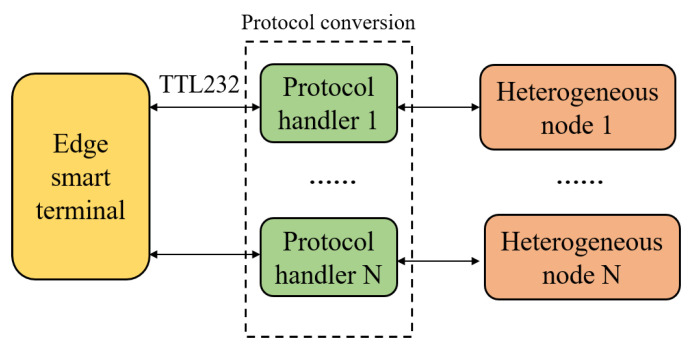
Heterogeneous nodes networking framework.

Figure 4 shows the heterogeneous nodes networking framework. The edge intelligent terminal uses a unified TTL232 interface to communicate with various heterogeneous network nodes. Since the communication interfaces of heterogeneous nodes are different, a coprocessor is introduced into the network to bridge the edge intelligent terminal, and various heterogeneous nodes complete the information exchange with the intelligent terminal through the coprocessor. For an example, a Bluetooth module is introduced for data collection at first, such as temperature data, and then these data are transmitted to the corresponding coordinators. Second, these coordinators transmit the data to coprocessors through the corresponding communication interface, such as SPI, IIC, etc. Finally, the coprocessors transmit the data to the intelligent terminal through a unified interface.

## 4. Materials and Methods

In order to realize remote calibrate work, we add some functional modules, such as wireless serial port module, Bluetooth module, XMPP module, Text recognition module, and so on.

### 4.1. Wireless Serial Port Module

The wireless serial port module is composed of a transmitter and a receiver. The transmitter consists of an ESP32 as the master single-chip microcomputer. The ESP32 is responsible for collecting information from sensors such as temperature sensors and cameras, and then driving the wireless serial port transmitter module to send the sensor information to the receiver. The received data will be transmitted in the form of a serial port protocol. The main control development board AIO3339J of the monitoring system integrates a serial port module, which can obtain the message sent by the sensors.

**Figure 5:**
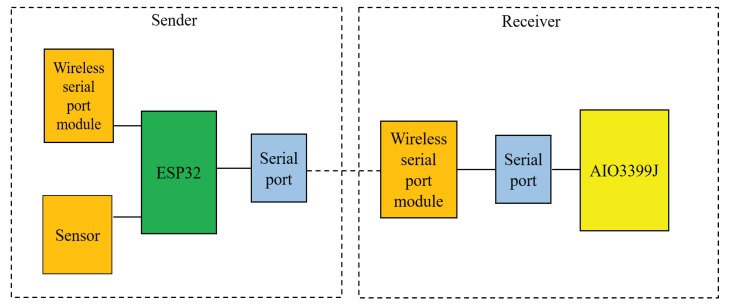
Schematic diagram of wireless serial communication.

Because Java cannot directly read and write to the serial port of the android client, we need to use c or c++ language to directly read and write the serial port of an Android device. Therefore, JNI is used to bridge the part of the serial port. This paper uses Google’s serial port communication interface -android-serial port-api to realize this function. The system block diagram is shown in Figure 5.

### 4.2. Bluetooth Module

**Figure 6:**
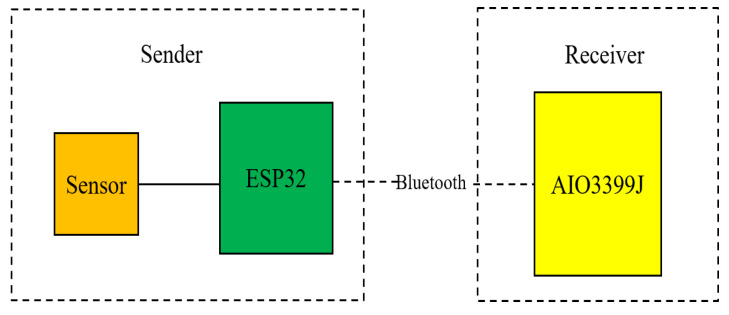
Bluetooth system framework communication.

Android integrates Bluetooth-related packages, and uses getDefaultAdapter() to obtain an AIO3399J Bluetooth adapter, and then uses startDiscovery() to search for nearby Bluetooth devices to complete the pairing to complete the Bluetooth communication. The system implementation block diagram is shown in Figure 6.

### 4.3. XMPP Module

XMPP uses a distributed network architecture. It is based on the Client/Server architecture [20]. XMPP uses Transport Layer Security (TLS) protocol as the encryption method of the communication channel in Client-to-Server communication and Server-to-Server communication. To ensure the security of communication, any XMPP server can be independent of the public XMPP network (for example, in the corporate internal network). This paper uses XMPP communication principles to implement hardware devices and servers. The communication is mainly manifested in the completion of message transmission through XMPP and finally transfers them to our database.

For the XMPP client, we use Smack. Smack is an open source, highly modular, and easy-to-use XMPP client library [21]. For the XMPP server, we use Openfire and Spark software to achieve message exchange.

### 4.4. Text Recognition Module

This part mainly realizes the automatic reading function of the electric meters to save the cost of manpower. It includes four principal steps: camera image capture, image text recognition, text analysis, and automatic data entry.

#### 4.4.1. Image Acquisition

We used the Android device to collect pictures from the electric meters in real time, and then call the camera of the Android device through the open-source library CameraKit to take pictures at regular intervals. We can also use the AIO3399J development board, one screen, and a camera to replace the Android device in experiments.

#### 4.4.2. Meter Recognition

Meter recognition belongs to the technical category of Optical Character Recognition (OCR). It is a science that enables translation of various types of documents or images into analyzable, editable, and searchable data [22]. Traditional OCR is based on image processing (binarization, connected domain analysis, projection analysis, etc., [23,24,25]) and statistical machine learning (Adaboot, SVM [26,27]) to extract the text content on the picture. However, traditional OCR performs poorly at character recognition in complex scenarios. Therefore, we use the deep learning approach to achieve text recognition. Text recognition is also divided into two steps, text detection, and text recognition.

- Text detection.

**Figure 7:**
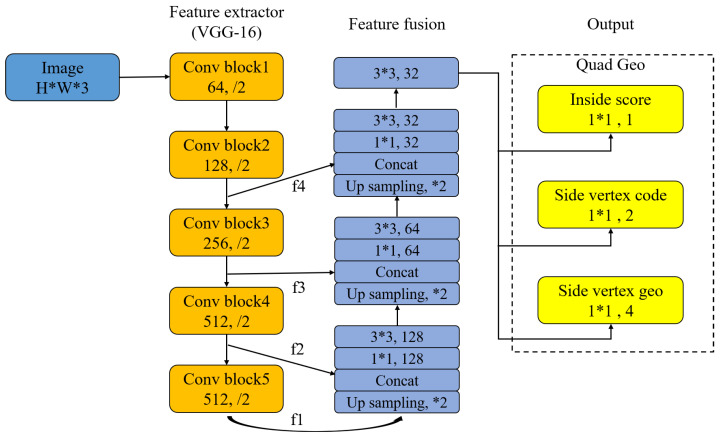
The network structure of FCN.

In this paper, we use the Efficient and Accurate Scene Text Detector (EAST) method, that is based on Fully Convolutional Networks (FCN), its network structure consists of three parts: feature extraction branch, feature fusion branch, and output layer. The feature extraction branch consists of a convolutional network, and this paper adopts the VGG-16 network structure [28], and the feature fusion branch uses the inverse pooling operation to obtain features of the same size as the convolutional feature map of the previous layer, and then splice with it and send it to the next convolutional layer. Finally, the output layer consists of two parts: one is the fractional map obtained with the convolution of a single channel, and the other is the geometric shape map obtained with the convolution of multiple channels, which can be a rotated box or quadrilateral. The network structure is shown in Figure 7.

- Text recognition.

The text recognition part adopts RCNN network structure: CNN + LSTM + CTC, where CNN is principally responsible for feature extraction of text.

In this paper, we built the simple Residual Network (ResNet) to extract image features. The core of ResNet introduces residual blocks on the basis of CNN [29]. We assume that the output function of an ordinary CNN is y=F(x), where *x* is the input, then, the output function after introducing the residual block becomes:

(1) y=F(x)+x

**Figure 8:**
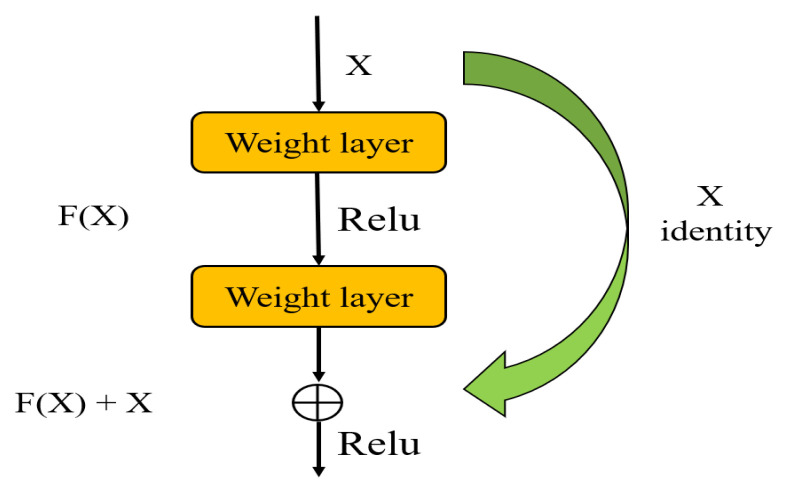
Simple residual block structure.

The structure of a simple residual block is shown in Figure 8.

Long Short Term Memory (LSTM) is introduced to prevent gradient disappearance or gradient explosion and solve the long-term dependency problem [30]. It is an improved model based on the Recurrent Neural Network (RNN).

Connectionist temporal classification (CTC) is used to solve the problem of misalignment of input features and output labels [31]. The core idea of the CTC algorithm is the introduction of blank symbols. Due to the different styles of the electric meters, the gap between each character in the training set image is different, so it is more appropriate for us to calculate the loss using the CTC method.

After text detection using EAST in the previous step, a rectangular box of the text region will be obtained. Then, we crop the original image according to the rectangular box region to obtain the input of the text recognition model (CNN + LSTM + CTC).

The CNN network extracts the features of the original image (height, width, channels) into a convolutional feature matrix of (N, T, D) as the input of the LSTM (2 layers), where N is the number of samples of the data input, T is the time of the LSTM, and D denotes the dimension of the input at each moment. For example, the original image (32, 100, 3) goes through three convolutional layers (convolution kernel size is 5), two pooling layers (pooling kernel size is 2), and the image size becomes the matrix of (1, 18, out-channels), so, T is equal to 18, D is equal to out-channels. Finally, for the output of the LSTM, we use CTC to calculate the loss.

**Figure 9:**
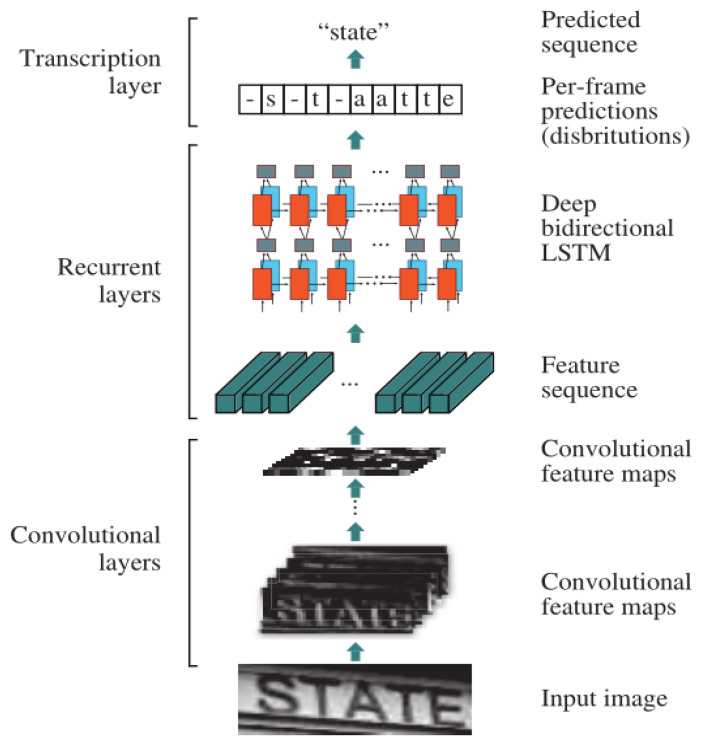
The framework of CNN + LSTM + CTC.

The CTC computational loss method is a good solution to the problem of misaligned input and output labels, and its training process is essential to adjust the LSTM model parameters by a gradient to maximize the output probability. The overall network structure of text recognition is shown in Figure 9.

#### 4.4.3. Text Parsing

Not all the content in the picture taken by the camera is useful. We only need to parse the useful recognized data and enter it into the database. For the same type of equipment or test the same type of data such as voltage, current, phase, etc., we can distinguish and analyze specific characters, such as “V”, “A”, “P”, and other characters based on the unit in the image.

### 4.5. Live Video Module

**Figure 10:**
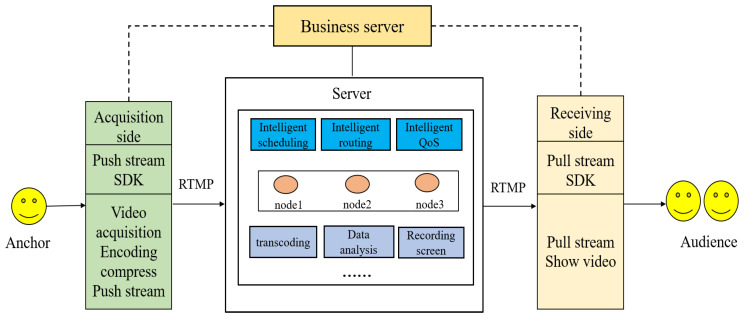
Live video framework.

This part realizes the real-time monitoring function of the remote calibration system. The live video module is composed of three parts: the push-stream SDK, the server-side SDK, and the pull-stream SDK. The entire system can be abstracted as the push end is the server of the live broadcast system, and the pull end is the client of the live broadcast system. In order to improve the efficiency of transmission, the server adopts the method of actively sending audio and video without the client sending a request, as shown in Figure 10. Since the processing power of the Android system is inadequate to support multiple video stream transmissions from the server to the client, a third-party server is added, and the video streaming is handled by the third-party server. This is the business server in the picture. There are currently a variety of servers on the market to choose from. Considering the demand and price, this paper selects the instant server as the business server.

The main function of the streaming SDK is to send audio and video data to the service server, using the RTMP protocol as a transmission tool. Among them, the push stream SDK is divided into audio and video acquisition modules, which are responsible for calling the camera to obtain the original camera data, the encoding compression module can ensure human visual and auditory effects while reducing the amount of data in the audio and video streams. The data are encapsulated according to the RTMP packet format. The server-side SDK needs to receive the audio and video streams transmitted by the push-side SDK. Then, we use the scheduling algorithm to transmit the audio and video streams to the responding node. The node here refers to the client connected to the business server. At the same time, the server-side SDK will also integrate flow calculation, data statistics, and recording and playback functions. The receiving end SDK needs to connect to the service server, and then receive the audio and video streams transmitted by the service server. At the same time, the receiving end SDK also needs to integrate the two modules of high-efficiency decoding and video display. High-efficiency decoding is equivalent to the reverse process of the previous audio and video stream compression, and finally the decoded audio and video stream is displayed on the android control.

## 5. Results

### 5.1. Part of Live Video

First, we initialize the SDK for streaming and pulling, establish a connection with the service server through this SDK. Then we create a room, set the room number and the ID of the audio and video stream. Finally, push the audio and video stream to the service server. The streaming end needs to initialize an SDK first, join the corresponding room, and pull the video stream corresponding to the ID in the room. In the app, a local preview is set on the push end, which corresponds to the screen on the left, and the pulled audio and video will be displayed on the screen on the right.

After testing, even in a weak network environment, this system still has good stability. It can guarantee a 100% entry into the room and successful push-pull streaming, without the phenomenon of failure of entry and push-pull streaming due to network packet loss. As shown in Table 2 and Table 3, the end-to-end delay does not exceed 600 ms in a weak network environment with 50% packet loss, and the delay can be controlled within 1000 ms in a weak network environment with 70% packet loss. The stability and real-time performance of this live streaming system ensure timely response when unexpected situations occur in monitoring scenarios.

### 5.2. Part of Text Recognition

For the specific scenario of text detection and recognition, the data samples are lightweight, and for the specific digital meter recognition in this paper, the types of characters are also lightweight. We compare the open-source datasets for text detection and recognition which are shown in Table 4, and we find that the EAST model can achieve good results in the specific scenario of this paper.

**Figure 11:**
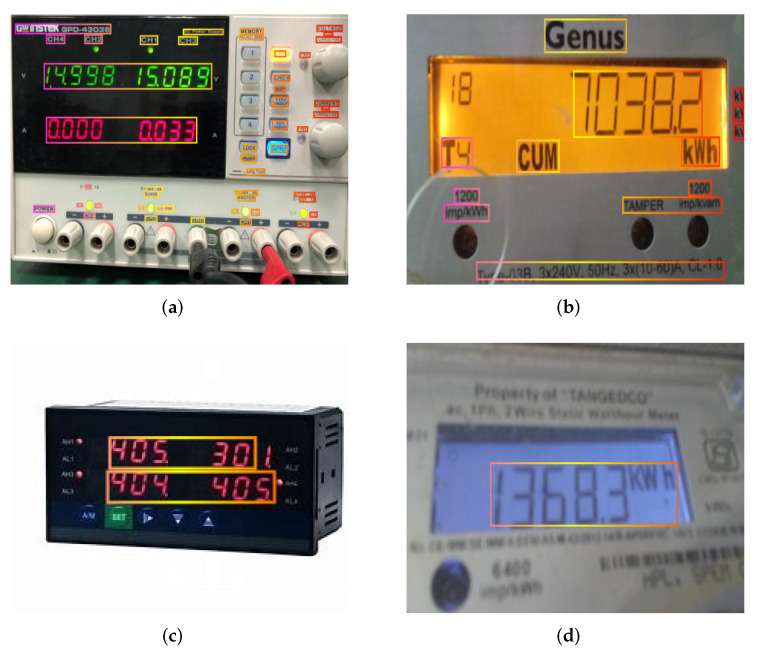
Part result of testing. (**a**) sample1, (**b**) sample2, (**c**) sample3, (**d**) sample4.

We selected some pictures of electric meters for testing, and the test results are shown in Figure 11.

For the scenario in this paper, we only need to recognize the meter data useful for calibration, so there is no need to recognize Chinese characters, and we only need to set a certain rectangular box area threshold to recognize the digital part of the meter, which reduces the complexity of the model and only supports the recognition of numbers and English characters. Some digital power equipment meters are collected on the Internet as the training set to train the model of text recognition.

**Figure 12:**
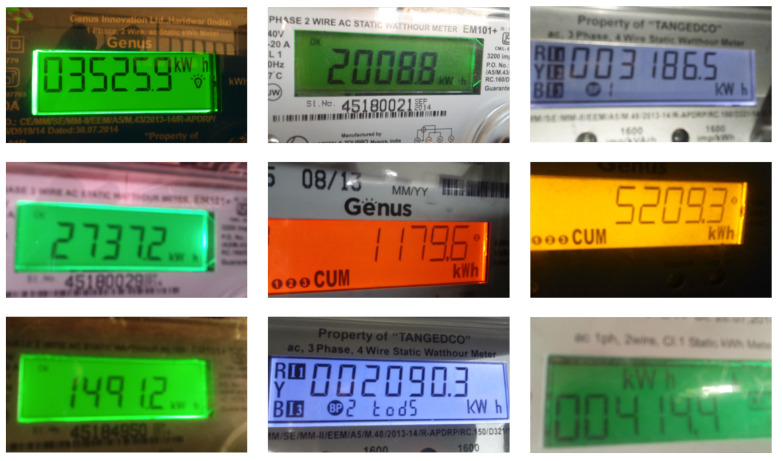
Some raw samples of our dataset.

**Figure 13:**
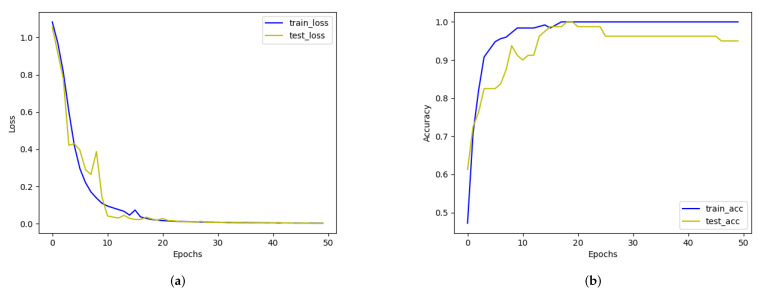
Process of model training. (**a**) Loss curve, (**b**) Accuracy curve.

Then, we build a CNN + LSTM + CTC model to train our dataset (some raw samples of our dataset are shown in Figure 12), and record the training process as shown in Figure 13. We set some hyper parameters as follows: learning rate is 0.001, epoch is 50, batch-size is 64, 8000 images for training and 2000 images for testing.

Ultimately, for the lightweight model in the specific scenario of this paper, good recognition results can be achieved, and the model metrics are shown in Table 5.

It should be noted that the data for these indicators are the result of taking the mean value.

### 5.3. Part of XMPP

**Figure 14:**
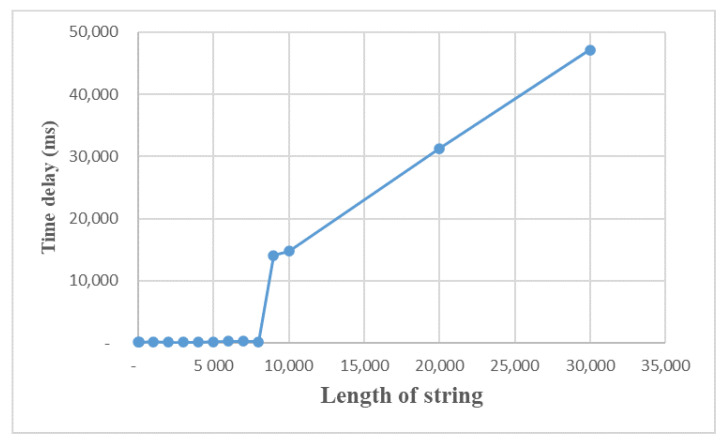
Testing of XMPP.

We test the message transmission effect with two XMPP accounts, as shown in Figure 14, and find that the delay is almost the same when the length of the string is below 8130, between 100 to 300 ms, and the sending time increases steeply to more than 14 s when the length exceeds 8130, and the delay is positively correlated with the length of the sent string.

The time delay depends on the type of transmitted message, such as character string, voice, picture, etc. Our current work is only limited to the string message type, in the future work, we will test other types of delay performance.

## 6. Discussion

The purpose of the work in this paper is to improve the intelligence of networking and informatization of remote calibration. In terms of networking, we use some edge devices with networking capability, and as the results in the paper show, the video calibration process has good performance in terms of delay and packet loss rate. In terms of informatization, we principally use technology related to artificial intelligence to realize image recognition and automatic data recording of instruments and meters.

We selected the EAST model for text detection because it was found to have good detection performance for text, and by observing the results of the study in Table 6, we decided to use the EAST model.

We selected the CRNN model (CNN + LSTM + CTC) for text recognition also because it was found that it showed good performance in text recognition and was suitable for various more complex scenarios, and by observing the results of the study in Table 7, we decided to use it.

In the actual recognition process, the biggest challenge encountered is that for the screen blurred instrument recognition effect is very bad, so our future major work is to reduce fog and noise of images.

In addition, in the actual calibration process, there will inevitably be data errors. There exists strict a data error range in standard calibration device, for example, the calibration index of lightning arrester is shown in Table 8.

In order to automatically detect the wrong data, one of our future jobs is to perform anomaly detection on the data of the measurement process.

## 7. Conclusions

With the development of smart instrument technology and Internet communication technology, we propose a remote calibration device that incorporates edge intelligence to improve the degree of intelligence and efficiency in remote calibration. The remote calibration scheme proposed in this paper uses Internet technology to combine software and hardware, and uses certain network protocols to connect various heterogeneous edge devices to complete the functional combination of modules. The live video module completes calibration monitoring well no matter if the network is good or bad. The text recognition module completes the recognition of meters and the experiment shows that our model can achieve over 90% recognition accuracy for some meters. The message transmission module completes the transmission of the data and information between the calibration laboratory and the central laboratory through XMPP. The overall remote calibration task is completed satisfactorily by using our remote device so that the calibration crosses the boundary of time and space, which can not only effectively reduce the cost of manpower but also improve the intelligence and efficiency of our remote calibration.

## Figures and Tables

**Table 1:** International existing remote calibration projects.

Institute	Remote Calibration Projects
NIST	measurement networks, electrical quantity calibration, high flow gas flow meters, etc.
FIPT	electronic calibration, AC Josephson Voltage reference, high pressure gas flow reference, etc.
NMIJ	temperature, pressure, optical frequency Coordinate measuring machine, radiation, etc.
NPL	Standard resistance, voltage remote calibration, network analyzer, etc.

**Table 2:** Network performance test.

Lossless Network	None	None
Up weak network	Up packet loss	30%, 50%, 70%
Up weak network	Up delay	200, 400, 700 (ms)
Down weak network	Down packet loss	30%, 50%, 70%
Down weak network	Down delay	300, 500, 1000 (ms)

**Table 3:** Room login and pull stream situation.

Packet Loss	Room Login	Stream Pulling
Up packet loss 30%	100%	100%
Up packet loss 50%	100%	100%
Up packet loss 70%	100%	100%
Down packet loss 30%	100%	100%
Down packet loss 50%	100%	100%
Down packet loss 70%	100%	100%

**Table 4:** Detection results in different datasets based on EAST.

Datasets	Accuracy (%)	Recall (%)	F-Measure (%)
ICDAR2013 [32]	88.0	74.0	81.0
ICDAR2015 [33]	83.27	78.33	80.72
Ours	93.3	87.5	88.0

**Table 5:** Model metrics.

Model Name	Train Loss	Test Loss	Train acc	Test acc	Recall	F1 Score
CNN + LSTM + CTC	0.1117	0.1122	0.9713	0.9343	0.932	0.9307

**Table 6:** Text detection results on ICDAR 2015 incidental text dataset.

Methods	Accuracy (%)	Recall (%)	F-Measure (%)
Zhang et al. [34]	70.8	43.0	53.6
SegLink [35]	73.1	76.8	75.0
EAST [33]	**83.3**	**78.3**	**80.7**
SSTD [36]	80.0	73.0	77.0
He et al. [37]	82.0	80.0	81.0

**Table 7:** Recognition results on ICDAR 2013.

Methods	Accuracy (%)
Bissacco et al. [38]	87.6
Jaderberg et al. [39]	81.8
CRNN [12]	**89.6**

**Table 8:** The calibration index of lightning arrester.

Items	Index Description
Current	Measuring range (0.1–50) mA
	Maximum allowable error ± (0.2% for reading + 2 μA)
Phase	Measuring range (0–90 degree)
	Maximum allowable error ±0.1 degree

## Data Availability

Not applicable.

## Abbreviations

The following abbreviations are used in this manuscript:

EAST	Efficient and Accurate Scene Text Detector
CRNN	Convolutional Recurrent Neural Network
CTC	Connectionist Temporal Classification
LSTM	Long Short Term Memory
FCN	Fully Convolutional Network
XMPP	Extensible Messaging and Presence Protocol
RTMP	Real Time Messaging Protocol
OCR	Optical Character Recognition
NIST	National Institute of Standards and Technology
FITP	Federal Institute of Physics and Technology
NMIJ	National Measurement Institute of Japan
NPL	National Physical Laboratory
